# Retraction: Jafer et al. Design of New Power Management Circuit for Light Energy Harvesting System. *Sensors* 2016, *16*, 270

**DOI:** 10.3390/s16111831

**Published:** 2016-10-31

**Authors:** Sensors Editorial Office

**Affiliations:** MDPI AG, St. Alban-Anlage 66, 4052 Basel, Switzerland

We have recently discovered that the majority of the text and figures in the title paper [1] is copied from a previously published thesis [2]. We consider this a serious breach of publication ethics and the paper will be marked as retracted. 

In addition, the second author, Paul Stack, had no knowledge of the submission. We have been unable to verify the identity of the third author, Kevin MacNamee, and University College Cork do not have a record of a former student or employee of that name. Contact details that the editorial office was provided for the second and third authors are not valid and they are not considered responsible for the preparation, submission or publication of [1]. 

We very much regret that this was not discovered during the review process and offer our apologies to readers of *Sensors*. 
